# DEBATE: Do interventions based on behavioral theory work in the real world?

**DOI:** 10.1186/s12966-019-0795-4

**Published:** 2019-04-25

**Authors:** Martin S. Hagger, Mike Weed

**Affiliations:** 1 Psychological Sciences and Health Sciences Research Institute, University of California, Merced, USA; 2 Faculty of Sport and Health Sciences, University of Jyväskylä, Jyväskylä, Finland; 3 School of Applied Psychology, Griffith University, Brisbane, Australia; 4 Health Psychology and Behavioral Medicine Research Group, School of Psychology, Curtin University, Perth, Australia; 5 Centre for Sport, Physical Education & Activity Research (SPEAR), Canterbury Christ Church University, Canterbury, UK

**Keywords:** Behavioural interventions, Health behaviour change, Efficacy, Effectiveness, Health outcomes, Implementation

## Abstract

**Background:**

Behavioral scientists suggest that for behavior change interventions to work effectively, and deliver population-level health outcomes, they must be underpinned by behavioral theory. However, despite implementation of such interventions, population levels of both health outcomes and linked behaviors have remained relatively static. We debate the extent to which interventions based on behavioral theory work in the real world to address population health outcomes.

**Discussion:**

Hagger argues there is substantive evidence supporting the efficacy and effectiveness of interventions based on behavioral theory in promoting population-level health behavior change in the ‘real world’. However, large-scale effectiveness trials within existing networks are relatively scarce, and more are needed leveraging insights from implementation science. Importantly, sustained investment in effective behavioral interventions is needed, and behavioral scientists should engage in greater advocacy to persuade gatekeepers to invest in behavioral interventions.

Weed argues there is no evidence to demonstrate behavioral theory interventions are genuinely effective in real world settings in populations that are offered them: they are merely efficacious for those that receive them. Despite behavioral volatility that is a normal part of maintaining steady-state population behavior levels creating the illusion of effectiveness, interventions fail in shifting the curve of population behaviors because they focus on individuals rather than populations.

Hagger responds that behavioral interventions work in the ‘real world’ in spite of, not because of, flux in health behaviors, and that the contention that behavioral theory focuses solely on individual behavior change is inaccurate.

Weed responds that the focus on extending the controls of efficacy trials into implementation is impractical, uneconomic and futile, and this has squandered opportunities to conduct genuine effectiveness trials in naturalistic settings.

**Summary:**

Hagger contends that interventions based on behavioral theory are effective in changing population-level behavior in ‘real world’ contexts, but more evidence on how best to implement them and how to engage policymakers and practitioners to provide sustained funding is needed. Weed argues for a paradigm shift, away from aggregative attempts to effect individual behavior change towards a focus on disrupting social practices, underpinned by understanding social and economic causation of the distribution and acceptance of behaviors in a population.

## Introduction

Given evidence for the prevalence and the human and economic burden of non-communicable diseases, behaviors that may contribute to the incidences of such diseases are of increasing academic, political and societal concern. We debate the extent to which interventions based on behavioral theory work in the real world to contribute to addressing these concerns. This arises from a live debate at the Annual Meeting of the International Society of Behavioral Nutrition and Physical Activity held in Hong Kong in June 2018. The debate reported in this article was conducted using the same format as the ‘live’ debate, our cases were each written independently then exchanged simultaneously, and the same process repeated for the responses. Once this process was completed, we authored the joint conclusion comprising points of agreement, and areas where we disagree.

## In favor: Martin S. Hagger - interventions based on behavioral theory do work in the real world

### Background

Given epidemiological research associating multiple chronic disease risk with participation in health-related behaviors [1–5], health organizations and stakeholders have sought to develop behavioral interventions that lead to practically-significant changes in these behaviors and concomitant reduction in disease risk. Behavioral scientists propose that interventions based on theories from the behavioral sciences, particularly psychology, will be optimally effective in evoking behavior change [6–13]. Despite an expanding evidence base demonstrating the efficacy of theory-based interventions in promoting sustained change in health-related behavior, my colleague will suggest that the role of behavioral theory is overstated, particularly when it comes to ‘real world’ effectiveness. In particular, he will argue that what works in ‘ideal world’ carefully-controlled conditions are not effective in ‘real world’ contexts where upscaling, logistic, cultural, and implementation factors pervade. He will cite the lack of change in physical activity participation and rates of chronic disease and obesity as evidence that interventions based on behavioral theory are not effective. Here I deconstruct these ‘straw person’ arguments, and contend that interventions based on theory can and do work in promoting behavior change in real world contexts.

### Do interventions based on theory ‘work’ in changing behavior?

Behavioral theory is a broad term for a set of pre-specified ideas or predictions aimed at explaining behavior [7, 14, 15]. Behavioral theories come from multiple disciplines (e.g., psychology, sociology, behavioral economics), and identify multiple determinants or mechanisms of behavior including beliefs, motivation and intentions [16], individual differences [17], social influence [18], and environment and demographics [19–21]. A substantive body of research has identified the effectiveness of theory-based interventions targeting change in modifiable determinants or mechanisms [22–24]. For example, syntheses of evidence have indicated that interventions targeting change in social cognitive beliefs and motivation [25–31], social support and norms [32, 33], and planning [29] to be effective in promoting behavior change in randomized controlled trials. Similarly, interventions based on health-risk communications have been successful in promoting behavior change [34], with graphic images on tobacco products a prominent example [35–38]. Research targeting change in determinants derived from social-ecological theories, encompassing environmental, community, and policy factors, have also been shown to be effective [21, 32, 39, 40]. Interventions based on choice architecture, sometimes referred to as ‘nudging’, have demonstrated effectiveness in changing behavior in laboratory and field settings [41–46]. In addition, interventions adopting specific strategies such as self-monitoring [47, 48], prompting social support [48], planning [49], behavioral skills [31], and affective appeals [50] have been found to be particularly effective. Taken together, primary studies and research syntheses indicate that theory-based interventions are effective in changing behavior in laboratory and ‘real world’ contexts [51].

In the interests of balance, it would be remiss not to acknowledge a number of caveats to this evidence. Meta-analyses and systematic reviews have also indicated that stated theoretical basis leads to no difference in intervention effectiveness [52], and, in some cases, even reverse effects [53]. Similarly, there is research demonstrating that adoption of particular behavior change strategies does not lead to greater intervention effectiveness [52, 54, 55]. So how can these two streams of evidence be reconciled? Inadequate mapping of theory on to intervention components may be a moderating factor. A distinction has been made between theory-inspired and theory-based interventions [56, 57]. Prestwich et al. [52] indicated that ‘theory-inspired’ interventions provide insufficient specification of links between theory and intervention strategies. Theory-inspired interventions, therefore, pay ‘lip service’ to behavioral theory, but fail to link intervention components with relevant theoretical determinants. There are also problems with inadequate reporting of such links, which hinders researchers’ ability to evaluate the effect of theoretical basis on intervention effectiveness. There is therefore a need for researchers to become more effective in matching theoretical determinants of behavior with intervention content, and for greater transparency when reporting intervention content [56, 58].

### Why have behavioral interventions not altered the course of non-communicable disease pandemics?

If interventions based on behavioral theory work in changing behavior in ‘real world’ contexts, how have they not stemmed the tide of non-communicable disease pandemics, as my colleague will contend? Knowledge and implementation of effective interventions, whether or not they are based on theory, seems to have had limited impact in changing population-level participation in health behaviors and reducing incidence of chronic disease [13]. Although there is substantive evidence that behavioral interventions are effective in changing behavior across multiple contexts, populations, and behaviors, and, arguably, those based on theory having greater effectiveness despite some of the aforementioned limitations, such knowledge is seldom translated to population-level change. This is largely because many behavioral interventions implemented at the community or even population level are relatively short lived, under-funded, or fail due to poor implementation, up-scaling, or translation [59]. Funding is a key issue; many behavioral interventions receive initial investment that is not sustained [60]. Interventions need sufficient funding for the necessary networks and providers required to implement the intervention in practice. Even though economic evaluation of many behavior change interventions has demonstrated their cost effectiveness [61], investment in behavioral interventions pales compared to investment in procedures aimed at treating disease [60]. It is unrealistic to expect health care providers to identify, assimilate, and implement research findings reported in scientific outlets. The onus is on those producing the evidence to actively engage governments, stakeholders and policymakers, and outline the human and economic advantages of preventive strategies like behavioral interventions over a treatment-focused model of healthcare provision [62].

Related to this, behavioral scientists need to better demonstrate how theory-based behavioral interventions that work in lab and field experiments, and have been shown to be effective in larger randomized controlled trials and in real world contexts, can be implemented in practice [9, 15, 63, 64]. Such evidence should be the focus of evidence presentations to government and policymakers advocating investment in, and implementation of, behavioral interventions [65]. The expanding discipline of implementation science focuses on translation of research findings into evidence-based practice, and is receiving increased attention in the fields of behavioral science, public health, health promotion, and health policy [65–67]. In the context of behavioral interventions, implementation science examines the pathways and strategies necessary for the uptake and implementation of interventions by policymakers and providers. Evidence on how behavioral interventions can be developed by key workers within existing networks, who will ultimately be responsible for implementing the intervention (e.g., health ministers, healthcare providers, school administrators and teachers, workplace health managers, community leaders, urban planners), and how users of the intervention (i.e., those whose behavior needs to change) can be involved in the implementation, is important to ensure that interventions are practically relevant and sensitive to the contextual and cultural characteristics of target populations [64, 68]. In addition, research on how theory-based behavioral interventions can be upscaled so their reach within target populations is maximized and the changes in health behavior and health outcomes promised by formative research realized [69]. Research is needed to identify the conditions necessary to up-scale behavioral interventions in real world contexts, including identifying the partnerships needed to fund, implement, monitor, and maintain interventions; engaging stakeholders to assess the feasibility and acceptability of implementing the intervention in the target community or setting [70]; assisting governmental agencies in developing multi-level and multi-sectorial plans to implement interventions; and developing ways to embed interventions in existing networks throughout development from inception to implementation [69].

### Conclusion

In conclusion, interventions based on behavioral theory have been shown to be effective in changing health behavior. However, there is still need for more research on interventions that systematically and precisely map intervention content with theoretical determinants, and the need for greater transparency in the reporting of intervention content and protocols. Arguments that such behavioral interventions do not work in the real world based on observations that pandemics of non-communicable disease continue to rise, and large scale interventions have not shifted population-level participation in health behavior, as my colleague contends, are specious and miss the point. The issue is not that interventions based on behavior theory do not work in changing behavior in ‘real world’ contexts, they do, rather, it is a lack of investment in, and inadequate upscaling and implementation of, these interventions that has failed to translate their efficacy into sustained, long-term change at the population level.

## AGAINST: Mike Weed - interventions based on behavioral theory do not work in the real world

Over 50 years positive population behaviors or health outcomes for nutrition and physical activity have fallen or flatlined globally, and in individual countries. Data shows: rising global obesity since 1975 [71], and in individual countries including England [72], Chile [73], and Australia [71]; falling or flatlining fruit and vegetable consumption in USA since 1994 [74], and in Japan [75] and Brazil [76] since 1965; and rising physical inactivity globally since 2001 [77], in Spain since 1995 [78], in USA since 1997 [79], and in China since 1989 [80]. For health outcomes, European Environment Agency data show loss of healthy life years attributable to non-communicable diseases has grown by more than 20% since 1990 [81]. These data illustrate global trends, and their replication in individual countries.

Something isn’t working! Noting disjoint between a body of behavioral theory literature that appears to show promise at the individual level, and global and national data that shows no change in population behaviors and health outcomes for half a century, Hallal et al. [82] argue “after more than 60 years of scientific research… more of the same (in terms of research and practice) will not be enough” (p. 190). It appears behavioral theory has a case to answer, and some fundamental questions to face. But is the problem scale-up of behavioral theory in population level interventions and policies, is it intervention designs that act as the vehicle for behavioral theory, or is it simply that behavioral theory itself does not work in the real world?

### Behavioral theory interventions are efficacious not effective

For decades fields such as exercise physiology, public health, epidemiology and the behavioral sciences have undertaken research showing that if behavioral theory is deployed “under scientifically controlled circumstances, behavior change is achievable for increasing physical activity” [69] (p. 1337). However, many “so-called effective physical activity interventions” (p. 1337) are small-scale, controlled efficacy trials that do not demonstrate effectiveness or ecological validity, and leave gaps in the chain of evidence between participants, theory, behavior and health outcomes [83]. An intervention is efficacious if it works in cohorts who receive it, whereas it is effective if it works in cohorts who have been offered it [84]. This is confused in the literature, and interventions based on behavioral theory claim effectiveness when available evidence demonstrates only their efficacy [69].

Many trials of interventions based on behavioral theory do not venture beyond controlled environments of phase I-III trials, which seek to establish, respectively, concept, efficacy and comparative efficacy. Thus, at best, evidence demonstrates that impact on those who receive the intervention exceeds impact on those who receive alternative interventions. But still, this shows only that an intervention is comparatively efficacious for those who receive it, not that it is effective, or comparatively effective, in cohorts that are offered it [84].

The problem is this: the features of design and implementation associated with good phase I-III trials to establish concept, efficacy, and comparative efficacy, have important limitations for informing practice and policy decisions [85], which require more generalizable information relating to outcomes of societal consequence, such as a sustained impact on health outcomes at population level. Such impact, or the potential for it, must relate to real world effectiveness “as evaluated in an observational, non-interventional trial in a naturalistic setting” [86]. To establish effectiveness, phase IV trials require a more diverse set of methods than those required to establish concept, efficacy and comparative efficacy in phase I-III trials, and must involve a diversity of settings, participants and deliverers [87]. However, in reviewing studies purporting to examine effectiveness of physical activity interventions in the real world (i.e., phase IV trials), Beedie, Mann and Jimenez [88] found that many still tried to adopt laboratory style methods and controls that would be impractical or uneconomic in real-world settings [89].

Some authors [69, 84, 90] have advocated the RE-AIM framework as a Phase IV tool to develop the effectiveness of interventions shown to be efficacious at phases I-III. But, with its focus on ensuring reach, adoption, implementation integrity, and maintenance of the features of the intervention over time, RE-AIM merely attempts to deliver effectiveness by maintaining the controlled environment of phase I-III trials in the real world, which as well as being impractical or uneconomic [89], is also likely to be futile.

Establishing effectiveness in phase IV trials is difficult, and requires longer timescales, and greater scale and resources than establishing concept, efficacy and comparative efficacy in phase I-III trials. As such, it is not surprising that, in an area where research funding is relatively sparse, and doctoral studies (which are time and resource limited) are often the bricks contributing to edifices of knowledge, genuine phase IV effectiveness trials are rare [89]. Nevertheless, there is a moral obligation to conduct them [87], otherwise advocacy for behavioral theory interventions based only on efficacy evidence risks wasting participants time (and hopes for their health) and taxpayers money on unproven interventions in unproven populations.

### Behavioral theory interventions are recipients not stimuli of behavior change

Analyses of national participation data suggest interventions based on behavioral theory may be recipients of individual behavior change, rather than the stimulus for it. This is because populations’ behaviors are qualitatively different to individual behaviors, and incorporate individual behavioral volatility within their steady state. For example, in England two national surveys, Active People (*n* = 150,000+) and Taking Part (*n* = 15,000+) [91, 92], show population participation in sport and related physical activity has flatlined for 10 years, with no sustained change beyond +/− 2%. Furthermore, data synthesis across six surveys shows falling or flatlining participation for 25 years [83]. However, both cross-sectional retrospective report data and panel time-series data from the surveys also shows considerable individual behavioral volatility, with circa 20% of the population dropping out or doing less sport, 20% taking up or doing more sport, 20% maintaining participation, and 40% consistently doing no sport [83]. Consequently, within any 1 year circa 40% of the population change their sport participation behavior, but aggregate population level participation is unchanged. Thus, steady state population behaviors incorporate considerable individual behavioral change. This suggests behavioral theory interventions are reflecting and facilitating individual behavior changes that take place as part of the steady state behaviors of populations, with participants often presenting as already motivated to change [88, 93]. Sport England’s Get Active: Get Healthy [94] first-year pilots, for example, claimed to be the stimulus for more than 30,000 people becoming active, but the evaluation showed the majority of participants were “ready to change” when they joined. This suggests the interventions were the recipients rather than the stimulus for individual behavioral changes, which are to be expected as a normal part of steady state population behaviors.

### Behavioral theory interventions are not linked to the causes of behavior

A health outcome stubbornly maintained in steady state population behaviors is widespread health inequality. It is known that poor health outcomes, particularly non-communicable diseases, correlate with social deprivation, low employment, poverty, poor housing, and other indices of multiple deprivation [95]. Behavioral theory provides neither the explanation nor, through interventions targeting individuals, the solution to such problems, which must focus on wider causal systems that underpin the social practice and economy of behaviors such as low physical activity and poor diet.

Undoubtedly, it is the focus on the individual rather than the population that undermines the real-world effectiveness of behavioral theory. The etiological model on which it is based – that poor health outcomes are caused by exposure to a substance, for example, sugar, and that health outcomes can be improved by modifying or moderating individual behaviors to remove or reduce exposure [96] – is fundamentally flawed. This is because solutions – interventions based on behavioral theory – have no relationship to causes – the factors that lead to behaviors in the first place. Furthermore, behavioral theory is assumed to be universal: that is, it is assumed the same behavioral theory can address any behavior, be that smoking, alcohol consumption, poor diet, or low physical activity – the transtheoretical model, which was developed for smoking cessation, is a case in point [97–100]. Cleary these behaviors are underpinned by different antecedents, so why would we assume they can all be addressed by the same theory? Furthermore, categories of behavior are not homogenous – the existence of health inequalities is, in itself, evidence that the factors that lead to behaviors in relation to, for example, diet, differ across the population, and so poor diet is an agglomeration of behaviors rather than a single behavior. Why would we expect that these multiple complex behaviors could all be addressed by the same theory?

### Conclusion

I have argued that while interventions based on behavioral theory have been shown to be efficacious in the controlled environments of phase I-III trials, there is no evidence from genuine phase IV effectiveness trials to demonstrate they work in the real world. However, crucially, I argue that evidence from controlled trials of behavior change interventions simply capture individual behavioral volatility that is a normal part of steady state population behaviors. Furthermore, such interventions fail in shifting population behaviors because they focus on individuals rather than on the multiple complex factors that drive the distribution of behaviors in the population. As such, behavioral theory within such interventions is not an active ingredient, rather it is a dormant recipient of behavior change. Put simply, behavioral theory has no active influence on changing behaviors in the real world.

## RESPONSE: Martin S. Hagger

I am grateful to my colleague for raising important points on the implementation of theory-based behavioral interventions and the need for more evidence for the effectiveness of behavioral interventions in ‘phase IV’ trials. These are good points that have been made many times elsewhere [64], including my opening statement. However, as an argument against the proposal, his statement is not fit-for-purpose. As I predicted, my colleague claims that interventions based on behavioral theory do not work in changing behavior in ‘real world’ contexts because there has been no year-on-year change in rates of non-communicable diseases and health-related behavior participation at the population level. He also suggests that behavior theory focuses solely on individual behavior, targets only the motivated, and fails to incorporate structural determinants of behavior. Here I illustrate how his arguments reflect a poor understanding of behavioral theory, and are not based on appropriate evidence, or, in some instances, any evidence at all.

My colleague’s argument that interventions based on behavioral theory do not work is flawed. He equates “behavioral interventions” with “theoretical basis”, and therefore claims theory-based interventions do not work because behavioral interventions have not been shown to work. As I argued in my opening statement, it is important to make a clear distinction between interventions based on behavioral theory, those merely ‘inspired’ by behavioral theory, and those that do not encompass theory at all [8, 56]. My colleague fails to make this distinction, and ignores evidence demonstrating the efficacy, and effectiveness, of interventions demonstrably based on behavioral theory in real world contexts [36, 41, 43, 101–103].

A further problem with his argument is to cite evidence of population-level non-change in rates of non-communicable disease and health-related behavior as evidence that behavioral theory does not work in the real world. It’s a poor argument without foundation. This is typified in his argument that the 25-year “falling or flatlining” of physical activity participation is somehow illustrative of a failure of behavioral interventions. This inference, which is speculative, is based on survey data on sport in which no intervention is identified. To make matters worse, this argument also infers that population-level changes in sport participation should reflect a desirable health-related outcome, a position he himself has argued against [83].

He also argues that considerable flux occurs in individuals’ behavior over time while a “steady state” is generally observed, suggesting that behavioral interventions capture this “volatility” rather than actual change. Yet, no evidence on theory-based behavioral interventions is offered to illustrate this point – the “Get Active: Get Healthy” campaign he cites, a sport-oriented intervention without basis in behavioral theory, provides no relevant data to verify this claim. Researchers designing trials of behavioral interventions are all too aware of the issue of time-dependent variability in health behaviors, and include appropriate covariates in their analyses to demonstrate intervention effectiveness is in spite of, not because of, population-level variation in behavior. However, better evidence than that cited by my colleague supports his contention that population level change in health behaviors has not been achieved [104]. Behavioral scientists’ should shoulder some of the blame for this failure by not advocating better implementation of effective interventions, but so too should all involved in the ‘chain of development’ of behavioral intervention from basic research to implementation.

My colleague also argues that: “Behavioral theory can provide neither the explanation…on the wider causal systems that underpin… behaviors such as low physical activity and poor diet”. This argument is incorrect, my colleague probably equates all behavioral theories as theories of individual behavior, which reflects a deficient knowledge of behavioral theory. Many behavioral theories incorporate socio-demographic, structural, and group-level variables as determinants of behavior, and propose how they interact with psychological determinants [21, 32, 39, 40]. Similarly, my colleague argues that: “Undoubtedly, it is the focus on the individual rather than the population that undermines the real-world effectiveness of behavioral theory.” I agree that a simple causal narrative, such as a sole focus on individual behavior change [96], will not be effective in reversing population-level incidence of non-communicable disease. However, this is not a failure of behavioral theory per se; many theories encompass individual, structural, and ecological determinants of behavior [105]. Rather, it points to a need to incorporate interventions based on behavioral theory into policy and practice through advances in implementation science [65–67].

Finally, my colleague suggests that theory-based behavioral interventions target only the motivated. This is not a new argument, intervention designers have been aware of this problem for years, and it is a problem that pervades mostinterventions, regardless of their theoretical basis. However, this is not the case for all interventions, and some of the most effective interventions work in changing behavior independent of motivation and in ‘real world’ contexts without the strict controls associated with laboratory research [36, 51, 102].

In conclusion, I commend my colleague for identifying the need for more effectiveness trials and translation efforts for theory-based behavioral interventions. However, his arguments against the effectiveness of behavioral theory in ‘real world’ contexts reflect an acute lack of understanding of behavioral theory, are based on incorrect inferences regarding behavioral theory, fall back on emotive language in an attempt to persuade, make no practical suggestions on the way forward for behavior change, and, as a consequence, should be summarily dismissed.

## RESPONSE: Mike Weed

The arguments for behavioral theory barely warrant rebuttal. The volume of evidence presented for effectiveness [25–51] demonstrates that those who receive behavioral theory interventions show changes in behavior compared to those who do not. This is evidence of efficacy, not effectiveness, a distinction not well understood in the literature. The appeal to implementation science is an attempt to extend controlled efficacy trial environments into implementation, focusing, for example, on maximising intervention fidelity [66] to achieve an illusion of effectiveness that is impractical, uneconomic and largely futile for achieving behavior change at scale. Finally, the litany of under-funded and unsustained interventions, which are presented as a result of poor communication by behavioral theorists and poor understanding by policy makers, suggests behavioral theorists are collaborating in their own victim narrative. Investment in implementing interventions has not been sustained because interventions have not been shown to be effective. But worst, because “implementation science” has sought to extend the controls of phase II and III efficacy and comparative efficacy trials into implementation, opportunities have been squandered to upgrade evidence by conducting genuine phase IV observational, non-interventional effectiveness trials in naturalistic settings.

### If not behavioral theory, then what?

Behaviors change regularly and often. In England, circa 18 million adults change their sport participation each year [91, 92], yet population sport participation levels have remained stable for quarter of a century [83]. Shifting the curve of population behaviors requires an entirely different approach than changing individual behavior. Successes in the former include the use of seatbelts, and reductions in drink-driving and in smoking in public spaces, but they result from legislative mandating, not effective behavioral theory interventions.

Since the 1960s in England, tobacco advertising, and then tobacco sponsorship, was regulated, restricted and then banned, followed by increasing restrictions and then a ban on smoking in public spaces, with a ban in cars and rented social housing now also being considered. Latterly, warnings then graphic images of increasing severity and size have been required on tobacco products, which now cover the whole packaging. Legislation has regulated messages and mandated behaviors, including mandated engagement with efficacious fear appeal interventions to ensure intervention fidelity and deliver effectiveness that would not otherwise be possible. Now, 50 years on, society no longer supports the social practice of smoking, and not only is the tobacco industry not permitted to reinforce smoking as a desirable behavior, it is required to undermine it. The role of behavioral theory in this process has been minimal; success is attributable to understanding the meanings attached to smoking as a social practice [106], the ways in which it is reinforced [107], and to addressing social and economic causation [96] through incremental legislative mandating [108] that disrupts the social practice of smoking.

### Is it time for a scientific revolution?

The academic practice of the development of behavioral theory shows the signs of paradigmatic science [109]. Theorists become self-reinforcing and self-referential devotees, advocates for theory rather than outcomes. Empirical deficiencies are attributed to imprecise specification or poor implementation, prompting calls for more meticulous use and more controlled implementation, or for tweaks at the margins of theory. Social ecological approaches, which co-opt social perspectives to support existing individualistic behavioral solutions, rather than to interrogate and understand social and economic causation, are an example of the latter. Kuhn [109] suggests these circumstances create the structure for scientific revolutions, in which empirical deficiencies can no longer be explained away at the margins or blamed on methods, and the old paradigm is displaced in favour of a new approach. I propose that new approach should be a social practice framework [96, 107, 109] that deploys legislative mandating as a tool to disrupt social practices, underpinned by understanding of social and economic causation. This should displace the current dominant individualistic behavioral paradigm that provides solutions that are not connected to causes. It’s time to burn down the house: the time for revolution is now!

## Joint conclusion

Although the current debate has showcased our different perspectives, it has also highlighted points of agreement. We both agree that interventions based on behavioral theory are efficacious in changing health-related behaviors. We also agree that there are problems with current evidence for the effectiveness of behavioral interventions, but we disagree on the nature and extent of these problems and their implications for drawing conclusions about the ‘real world’ effectiveness of behavioral theory. Beyond this, we also disagree on the implications of the evidence base as it stands for advancing effective, long-term solutions to the increasing prevalence of non-communicable diseases.

### Hagger

While evidence for real world effectiveness of interventions based on behavioral theory applied in real world contexts is limited, it is not absent. Good examples of theory-based interventions that have demonstrable real-world effectiveness in changing behavior exist (e.g., graphic warnings on tobacco products). Behavioral interventions offer a range of strategies that, if appropriately implemented, can and will make lasting changes in behavior at the population level. However, I recognize the need to develop the evidence base of effective large-scale behavioral interventions that can be embedded within existing networks, and are sensitive to the social and cultural norms of the target population. The interventions need to be sustainable through, for example, their incorporation into routine care or standard practice. Those developing interventions need to actively engage and lobby policymakers and governments to invest in interventions with demonstrated effectiveness and include them as core components of existing services. Behavioral interventions should be an integral part of a co-ordinated set of strategies that also includes policy change and legislation targeting change in specific behaviors at the population-level.

### Weed

Fundamental change is required: a paradigm shift to focus on social practice rather than individual behavior. Evidence that behavioral theory interventions are genuinely effective among those offered them, rather than simply efficacious among those receiving them, is all but absent, and absence of evidence is evidence of absence. The effectiveness gap is one of engagement that cannot be bridged by persuasion, rather mandating is required, either through legislation, or through interventions with mandatory engagement, such as point of choice information. Nonetheless, there is a role for behavioral theory: firstly, in providing efficacious support for individuals wishing to change; secondly, as a minor dimension of a social practice approach, which places historic and contemporary social and economic forces that lead to the existence of social practices, and that sustain them, at the centre, rather than the contemporary behaviors of individuals. Social practices can be disrupted over time through the incremental interplay of legislative mandating, and social change that creates the conditions for legislation. However, the circumstances and pace of disruption are rooted in understanding social and economic causation, and how this underpins the distribution and acceptance of behaviors in a population, not in aggregative attempts to effect individual behavior change.
